# Health-related quality of life and physical well-being among a 63-year-old cohort of women with androgenetic alopecia; a Finnish population-based study

**DOI:** 10.1186/1477-7525-3-49

**Published:** 2005-08-24

**Authors:** Päivi Hirsso, Ulla Rajala, Mauri Laakso, Liisa Hiltunen, Pirjo Härkönen, Sirkka Keinänen-Kiukaanniemi

**Affiliations:** 1 Department of Public Health Science and General Practice, Box 5000, FIN-90014 University of Oulu, Finland; 2 Unit of General Practice, Oulu University Hospital, FIN-90029 OYS, Finland; 3 Oulu Health Center, Box 8, FIN-90015 City of Oulu, Finland; 4 Oulu Deaconess Institute Department of Sports Medicine, Finland

**Keywords:** androgenic alopecia, hair loss, insulin resistance, health related quality of life

## Abstract

**Background:**

The aim of this study was to assess the possible associations between female androgenetic alopecia (AGA), insulin resistance and health-related quality of life (HRQOL)-linked factors in women. We hypothesized that not only the mental aspects but also certain physical aspect of women's health, such as insulin resistance, have an important role in the determination of HRQOL among women with hair loss.

**Methods:**

A population-based cohort of 330 healthy women aged 63 years, who participated in this study in the City of Oulu in Northern Finland, underwent a medical check-up including assessment of hair status on Ludwig's scale. Background data were collected with a standard questionnaire including a validated RAND 36-Item Health Survey (RAND-36) questionnaire.

**Results:**

105 (31%) women with AGA and 225 (69%) controls completed the RAND-36 questionnaire. The women with AGA were more insulin-resistant than the women with normal hair (QUICKI 0.337 vs. 0.346, p = 0.012). Impaired glucose regulation (IGR) was more prevalent among the former than the latter group (39% vs. 25%). The mean RAND-36 scores were significantly lower on the dimensions of physical functioning, role limitation due to physical health and general health, but not on the mental or social dimensions, among the women with AGA compared with the controls. In multivariate logistic regression analyses with the lowest quintiles of the HRQOL dimensions as the dependent variables and AGA, depression, marital status, education and IGR or QUICKI as independent variables, AGA was independently associated with role limitations due to physical health (2.2, 95% CI 1.20–4.05, 2.45 95% CI 1.32–4.55, respectively).

**Conclusion:**

In women aged 63 years, AGA was associated with role limitations due to physical health. Furthermore, the prevalence rates of IGR and insulin resistance measured by QUICKI were higher among the women with hair loss than those with normal hair.

## Background

Androgenetic alopecia (AGA) has been observed in women, with an increasing prevalence particularly after the age of 50 years [1]. Moreover, it has been suggested that hair loss could have an adverse effect on psychosocial life and self-esteem among both genders [2-7]. A number of studies have shown that, in selected clinical populations, androgenic alopecia is associated with poorer quality of life scores on disease-specific quality of life questionnaires [8,9]. Women with AGA describe their self-perceived health and psychosocial situation more negatively (e.g. low self-esteem, emotional distress due to AGA, social anxiety) than those with normal hair [2,5,8,9]. In addition, one study suggested that hair loss in women could be associated with psychiatric disorders, e.g. depression [3].

It is unclear why women with AGA would have a poorer quality of life. As most studies have looked at these associations in subgroups of women with underlying illness, it is plausible that the women with AGA may have an underlying subclinical disease. In males, an association between AGA and insulin resistance has been demonstrated [10,11], but the data on women are less conclusive [12].

We explored the association between AGA and various dimensions of HRQOL among community-dwelling women. We further explored whether any differences in the quality of life between those with and without AGA could be explained by an underlying metabolic abnormality. The aim of this study was to assess the potential associations between AGA, insulin resistance (IR) and the different HRQOL dimensions among women at the population level.

## Methods

A population-based study was carried out in northern Finland in 1990–1992 to assess, among other things, the prevalence of diabetes mellitus (DM) and impaired glucose tolerance (IGT). Altogether 831 subjects (435 women) born in 1935 and living in Oulu, a city with 100,000 inhabitants, participated in the study. They were invited to attend a follow-up study in 1996–1998, and 593(71%) subjects (347 women, 83%) attended. A detailed description of the data has been published earlier [13].

As part of the clinical examination, hair status (330 women, 93% of those participated) was assessed by the same trained nurse, using Ludwig's scale [14], and re-classified into two classes for the analysis. The original classes 0 and I (no or minimal hair loss) were combined as a normal hair group and the original classes II and III (moderate or marked hair loss) were combined as a hair loss group.

We measured health-related quality of life (HRQOL) using the Finnish version of the RAND 36-Item Health Survey 1.0 (RAND-36) [15]. This self-reported measure is composed of eight separate scales assessing physical functioning (10 items), role limitations due to physical health (4 items), role limitations due to emotional problems (3 items), energy/fatigue (4 items), emotional well-being (5 items), social functioning (2 items), pain (2 items) and general health (5 items). All scale scores range from 0 to 100, with 100 representing the most favourable functioning/well-being, and the minimal clinically important difference is cautiously suggested to be 3–5 points [16,17]. RAND-36 [18] includes the same items as the Item Short-Form Health Survey SF-36 [19], but the scoring algorithm has been slightly modified [18].

From the questionnaires, we assessed various sociodemographic variables, including the respondents' current marital status (married, unmarried/divorced or widowed) and educational attainment (elementary, secondary or university). Self-perceived health and the participant's own opinion of his or her overall physical fitness and life satisfaction were also asked (good, moderate or poor). A sum score of overall physical capacity was constructed based on their ability to walk up one flight of steps, a few flights of steps, half a kilometre or two kilometres or to run one hundred metres. The total score ranged from 0 to 20, with a higher score indicating poorer physical capacity. Depressive symptoms were measured using the Beck Depression Inventory (BDI) [20] with scores ≥ 10 defined as indicative of depressive symptoms.

Anthropometric measurements (weight, height, waist circumference and hip circumference) were obtained and used to calculate body mass index (weight/height^2^) (BMI) and waist-to-hip ratio (WHR). Blood pressure (BP) was measured by a physician from both arms in a sitting position. The mean value of these two measurements was used in the analyses. Hypertension was defined as either a systolic blood pressure ≥ 160 mmHg or a diastolic blood pressure ≥ 90 mmHg or being on antihypertensive medication regardless of the blood pressure values. Prevalent chronic disease (hypertension, ischemic heart disease, diabetes, stroke, intermittent claudication, arthritis) was based on a medical diagnosis as reported by the participant on the questionnaire or the use of medication for any of these diseases as reported during the clinical examination.

After an overnight fast, blood samples were collected using a standardized procedure and assayed for blood glucose (fB-gluc), two-hour blood glucose concentration (2 h-gluc) and serum insulin (excluding diabetic patients with insulin treatment). A standardized 75-g oral glucose tolerance test (OGTT) was performed. We defined the participants as having impaired glucose regulation (IGR) if they met the criteria for having impaired fasting glucose (IFG), IGT and DM based on the WHO 1997 criteria [21]. To measure insulin sensitivity, a quantitative insulin sensitivity check index (QUICKI) was used [22,23]. QUICKI was determined from the fasting insulin and glucose values according to the equation: QUICKI = 1/ [log(fasting insulin) + log(fasting glucose)]. Diabetic subjects with antidiabetic medication were excluded from the analysis.

### Statistical analyses

For these analyses, we used data from 347 women with complete hair status scores (n = 330). Descriptive comparisons of the groups defined by hair status were presented as cross-tabulations and percentages for the categorical variables and assessed with the T-test when the distribution was normal or with the Mann-Whitney U-test in the case of a non-normal distribution of the continuous variables. The statistical differences between the scores of the HRQOL dimensions were tested by the Mann-Whitney U-test [24]. The HRQOL dimensions on which the women with significant hair loss scored lower than the women with normal hair were analyzed in more detail. After bivariate comparisons, multivariate logistic regression analyses with the lowest quintile of HRQOL as the dependent variables and AGA, BDI, IGR/QUICKI, marital status and education as independent variables were made. The possible interactions of hair status with depression (AGA*BDI) and impaired glucose regulation with depression (IGR*BDI) were tested by interaction terms.

## Results

The prevalence of moderate to marked hair loss (grade II-III on Ludwig's scale) was 31% among the study population. Table 1 gives the percentages and Table 2 gives the means of the background characteristics of the women stratified into the categories of normal hair (grade 0 and I on Ludwig's scale) and hair loss (grades II and III on Ludwig's scale). In bivariate analysis, the women with hair loss had statistically significantly lower self-perceived health, self-perceived physical fitness and life satisfaction compared to the women with normal hair. (Table 1) Waist circumference and WHR were significantly higher and BMI tended to be higher (p = 0.094) among the women with hair loss compared with those with normal hair status. The mean QUICKI was lower, indicating higher IR, in the women with hair loss compared with the women with normal hair. (Table 2) The sum score of overall physical capacity was significantly higher (describing the limitation in physical functions) among the women with hair loss compared with the women with normal hair (mean sum score 7.9 ± 3.1 vs. 7.2 ± 2.8, p = 0.015).

**Table 1:** Percentages of some background characteristics among the study groups

Variable	**Hair loss (n = 105)**	**Normal hair (n = 225)**	
	% (n)	% (n)	p
Marital status			0.163
married	59 (61)	69 (154)	
not married/divorced	27 (28)	18 (41)	
widow	14 (14)	13 (29)	
Educational level			0.115
elementary	89 (92)	80 (181)	
secondary	5 (5)	11 (25)	
tertiary	6 (6)	8 (19)	
Self-perceived health			0.030
good	41 (43)	54 (121)	
moderate	48 (50)	42 (94)	
poor	11 (11)	4 (10)	
Self-perceived physical fitness			0.001
good	26 (27)	46 (104)	
moderate	54 (56)	44 (99)	
poor	20 (21)	10 (22)	
Life satisfaction			0.044
satisfied	66 (69)	79 (176)	
moderately satisfied	32 (34)	20 (45)	
dissatisfied	2 (2)	1 (3)	
BDI^1)^			0.311
no depression	76 (78)	83 (173)	
depressive symptoms	22 (21)	17 (35)	

^1) ^BDI = Beck's Depression Inventory; cut point ≥ 10

**Table 2:** Mean and standard deviations (SD) of background characteristics among women with hair loss and normal hair

Variable	**Hair loss n = 105**		**Normal hair n = 225**		
	
	Mean	Sd	Mean	Sd	p
Waist circ. (cm)	86.6	11.4	83.0	10.9	0.010
BMI ^1) ^(kg/m^2^)	28.5	4.5	27.5	4.0	0.094
WHR^2) ^(waist/hip)	0.84	0.06	0.81	0.06	0.002
fB-gluc^3) ^(mmol/l)	5.2	1.0	5.0	0.8	0.064
2-h gluc^4) ^(mmol/l)	7.2	1.8	7.1	1.8	0.321
QUICKI^5)^	0.337	0.03	0.346	0.03	0.012

^1) ^BMI = body mass index (kg/m^2^); ^2) ^WHR = waist to hip ratio; ^3) ^fB-gluc = fasting glucose level (mmol/l); ^4) ^2-h gluc = 2-h blood glucose level (mmol/l); ^5) ^QUICKI = Qualitative Insulin Sensitivity Check Index

There were no significant differences between the hair status categories of the women reporting heart failure, hypertension or musculoskeletal symptoms, but the women with hair loss self-reported more DM than those with normal hair (14% vs. 6%). The corresponding prevalences for IFG and IGT were as follows (2% vs. 5%, 24% vs.16%, respectively). The prevalence of IGR (cluster of self-reported DM and IFG, IGT and DM according to OGTT) defined by WHO was different between the groups (43% vs. 30%, p = 0.015).

The women with hair loss scored significantly lower on the physical functioning, role limitations due to physical health and general health perceptions dimensions of HRQOL compared with the women having normal hair. Also, a nearly statistical significant difference was seen in the pain score between the groups. (Table 3).

**Table 3:** Means/medians and standard deviation (SD) / interquartile range of HRQOL dimensions of women with hair loss and normal hair

Scale	**Hair loss (n = 105)**		**Normal hair (n = 225)**		
	
	Mean	SD	Mean	SD	p
Physical functioning	71,2	20,7	77,9	19,4	0,004
Energy/fatigue	69,0	19,3	70,4	17,9	0.431
Emotional well-being	78,9	15,5	78,7	16,4	0.557
Social functioning	85,7	19,3	88,7	18,5	0.170
Pain	68,6	24,7	73,2	24,9	0,085
General health	55,9	17,3	60,8	16,8	0,007
					
	Median	Q1–Q3	Median	Q1–Q3	
		
Role limitations due to physical health	75	25–100	100	50–100	0,032
Role limitations due to emotional problems	100	33–100	100	67–100	0,350

The interaction terms of depression with hair loss and depression with IGR were not statistically significant, suggesting that the effects of hair loss and IGR on the dimensions of HRQOL were not different in depressive and non-depressive subjects. The multivariate logistic regression analyses were made to analyze the independent association of AGA with the HRQOL dimensions. Two different adjusted models are presented in Table 4. In model a), glucose status is presented as IGR, and in model b), glucose metabolism is described with QUICKI. Because depressive symptoms, marital status and education are associated with HRQOL, they were included in the models as confounding factors. In both models AGA was independently associated with role limitations due to the physical health dimension of HRQOL, the odd rations being 2.2 (1.20–4.05) in model a) and 2.45 (1.32–4.55) in model b). In the dimensions of physical functioning and general health, no such association between AGA and HRQOL was seen. In addition, depressive symptoms were independently associated with role limitations due to physical health and general health.

**Table 4:** Multivariate logistic regression analyses for impaired quality of life. Odds ratios for risk of belonging to the lowest quintiles in the dimensions of quality of life

Variable	**Physical functioning**	
	Model a	Model b
	OR (% CI)	OR (% CI)
BDI^1)^	1.96 (0.98–3.92)	2.21(1.10–4.44)
IGR^2)^	2.30 (1.29–4.25)	
QUICKI^3)^		2.60 (1.33–5.09)
AGA^4)^	1.47 (0.79–2.71)	1.58 (0.85–2.95)
Marital status	0.78 (0.28–2.17)	0.78 (0.28–2.21)
Education level	2.39 (0.88–6.53)	0.44 (0.59–1.20)
	**Role limitations due to physical health**	

	Model a	Model b
BDI^1)^	4.81 (1.20–4.05)	5.36 (2.75–10.6)
IGR^2)^	1.79 (0.99–3.24)	
QUICKI^3)^		1.15 (0.55–2.38)
AGA^4)^	2.20 (1.20–4.05)	2.45 (1.32–4.55)
Marital status	1.24 (0.45–3.48)	1.20 (0.42–3.44)
Education level	0.63 (0.30–1.33)	1.58 (0.74–3.35)
	**General health**	

	Model a	Model b
BDI^1)^	4.74 (2.42–9.31)	5.67 (2.84–11.3)
IGR^2)^	1.68 (0.89–3.17)	
QUICKI^3)^		1.62 (0.76–3.45)
AGA^4)^	1.58 (0.82–3.03)	1.83 (0.93–5.59)
Marital status	1.22 (0.55–3.41)	1.20 (0.34–3.79)
Education level	1.37 (0.55–3.41)	0.75 (0.29–1.20)

Model a; adjusting without QUICKI. Model b; adjusting without IRG.

^1) ^BDI = Beck's Depression Inventory; depressive symptoms ≥ 10.

^2) ^Impaired glucose tolerance according to WHO 1997 criteria.

^3) ^Insulin sensitivity check index; the lowest quintile.

^4) ^Androgenetic alopecia; classes II–III

## Discussion

The main finding of this study was that women with AGA had impaired self-perceived health. They were more insulin-resistant when measured by QUICKI as a marker of insulin sensitivity, and impaired glucose regulation was more prevalent among them compared with the women with normal hair. The women with AGA had significantly higher waist circumference and WHR values as markers of abdominal obesity than the women with normal hair. Compared with the women having normal hair, the women with AGA scored significantly lower on three of the eight RAND-36 dimensions: physical functioning, role limitations due to physical health and general health items compared with the women having normal hair. In addition, the scores on all the other RAND-36 dimensions except emotional well-being tended to be lower.

This is the first study of an unselected and representative population of women to report an association of AGA with the general measures of HRQOL, which are recommended for epidemiological studies. The results of the previous studies concerning the quality of life of AGA subjects are somewhat difficult to compare with our results because of methodological and other differences, such as age, limited criteria of quality of life and the selection of study populations [5,8,9]. Actually, the previous studies by Schmidt and co-workers, in which quality of life was measured with an instrument specific for patients with hair loss (Hairdex), showed reduced values in the social and emotional domains in female patients with alopecia [8,9]. Only experiences of negative psychological effects on their quality of life have been reported by women with hair loss [5].

These results are opposite to our result, which might be explained by population differences and differences in the evaluation of HRQOL. Moreover, the women in our study were not seeking treatment for hair loss. The scaling properties and the validity of the Finnish RAND-36 have been tested among randomly selected Finns (3000 subjects aged 18–79 years and 400 subjects aged 65–79 years) [15]. The results were comparable with the results obtained for RAND-36 in international studies, but in the Finnish study, the general health scale correlated more strongly with physical health. In our study, all scores of dimensions were at the same level in this age-group as in the Finnish RAND-36 study, but differences were seen in comparisons of hair status.

The women with AGA were more insulin resistant, and IGR was more prevalent among them compared to those with normal hair. Moreover, central obesity, which was prevalent among women with AGA, is known to be associated with IR, type 2 diabetes mellitus, hypertension, dyslipidemia and coronary heart disease [25]. In our earlier studies, hair loss in 63-year-old women was suggested to be associated with insulin-linked disturbances, such as overweight, hyperinsulinemia and microalbuminuria [12]. A similar association has also been reported in men [10,11,26,27]. Furthermore, an association between depression and insulin resistance has been suggested among this study population [28]. This raises a question about the role of somatic diseases at the background of impaired HRQOL in women with AGA.

It is hence not self-evident that women with AGA have only emotional problems [2,3,5], but they may also have physical problems as well as chronic diseases and risk factors that need to be evaluated. According to our results, the physical dimensions, but not the mental health dimensions, have an important role in the determination of HRQOL in women with hair loss. In this study, the prevalence of depressive symptoms was not significantly higher in women with hair loss compared with women having normal hair. However, depression seemed to be a significant risk for lower HRQOL on the dimensions of physical health, role limitations due to physical health and general health in this cohort, which is in accordance with earlier studies [18,29]. This finding might be explained by the similarities in the measurement of psychological dimensions of HRQOL and BDI.

One limitation of our study may be that the hair classification was done by combining grade 0 and grade I (minimal hair loss). The reason is that they are difficult to differentiate from each other, especially in Finnish subjects with light and thin hair. Moreover, the classes II and III were combined because of the low number of women in class III (n = 4). The strengths of our study include the large and representative population sample of one female age group, giving the study the relevance of an epidemiologic survey, and the standardized scale used by the same trained nurse as part of the clinical examination, which means that inter-rater variation was lacking. We used well-documented questionnaires (RAND-36) to measure quality of life, which is a multidimensional phenomenon that includes several aspects of well-being. RAND-36 has well-documented reliability and validity and is useful in describing HRQOL in epidemiological studies of unselected populations [30].

## Conclusion

It can be concluded that AGA among 63-year-old women was associated with the physical aspects of HRQOL, but not with the social or mental health aspects. Hair loss was independently associated with role limitations due to physical health. In this study, AGA was not associated with the psychosocial aspects of HRQOL or depression. Instead, AGA was associated with IR, IGT and central obesity. Because these conditions are linked with somatic diseases, they can explain this finding of an association of AGA with impaired physical function. Hair loss could be a marker of poorer physical health, and women developing female AGA might benefit from attention to cardiovascular diseases and their risk factors in medical check-ups.

## Authors' contributions

PH conceived the study, reviewed the literature, analyzed the data and wrote the initial and subsequent drafts. UR, ML, LH and PiH collected the data and contributed to the study design, interpretation and revisions of the manuscript. SKK helped to conceive the study and revise the initial and subsequent drafts and supervised the research group. All authors read and approved the final manuscript.
